# Acetylcholinesterase inhibition in electric eel and human donor blood: an in vitro approach to investigate interspecies differences and human variability in toxicodynamics

**DOI:** 10.1007/s00204-020-02927-8

**Published:** 2020-10-10

**Authors:** Emma E. J. Kasteel, Sandra M. Nijmeijer, Keyvin Darney, Leonie S. Lautz, Jean Lou C. M. Dorne, Nynke I. Kramer, Remco H. S. Westerink

**Affiliations:** 1 Toxicology Division, Faculty of Veterinary Medicine, Institute for Risk Assessment Sciences (IRAS), Utrecht University, P.O. Box 80.177, 3508TD Utrecht, The Netherlands; 2 Risk Assessment Department, French Agency for Food, Environmental and Occupational Health and Safety (ANSES), 14 Rue Pierre et Marie Curie, 94701 Maisons-Alfort, France; 3 European Food Safety Authority, Scientific Committee and Emerging Risks Unit, Via Carlo Magno 1A, 43126 Parma, Italy

**Keywords:** Acetylcholinesterase, Pesticides, Human variability, In vitro toxicodynamics, Uncertainty factor

## Abstract

In chemical risk assessment, default uncertainty factors are used to account for interspecies and interindividual differences, and differences in toxicokinetics and toxicodynamics herein. However, these default factors come with little scientific support. Therefore, our aim was to develop an in vitro method, using acetylcholinesterase (AChE) inhibition as a proof of principle, to assess both interspecies and interindividual differences in toxicodynamics. Electric eel enzyme and human blood of 20 different donors (12 men/8 women) were exposed to eight different compounds (chlorpyrifos, chlorpyrifos-oxon, phosmet, phosmet-oxon, diazinon, diazinon-oxon, pirimicarb, rivastigmine) and inhibition of AChE was measured using the Ellman method. The organophosphate parent compounds, chlorpyrifos, phosmet and diazinon, did not show inhibition of AChE. All other compounds showed concentration-dependent inhibition of AChE, with IC_50_s in human blood ranging from 0.2–29 µM and IC_20_s ranging from 0.1–18 µM, indicating that AChE is inhibited at concentrations relevant to the in vivo human situation. The oxon analogues were more potent inhibitors of electric eel AChE compared to human AChE. The opposite was true for carbamates, pointing towards interspecies differences for AChE inhibition. Human interindividual variability was low and ranged from 5–25%, depending on the concentration. This study provides a reliable in vitro method for assessing human variability in AChE toxicodynamics. The data suggest that the default uncertainty factor of ~ 3.16 may overestimate human variability for this toxicity endpoint, implying that specific toxicodynamic-related adjustment factors can support quantitative in vitro to in vivo extrapolations that link kinetic and dynamic data to improve chemical risk assessment.

**Electronic supplementary material:**

The online version of this article (10.1007/s00204-020-02927-8) contains supplementary material, which is available to authorized users.

## Introduction

Acetylcholinesterase (AChE) is an important enzyme in the nervous system and a common target of toxicity. AChE hydrolyses the essential neurotransmitter acetylcholine (ACh), thereby preventing overstimulation of the ACh-receptor at the postsynaptic target cell (Soreq and Seidman 2001). Several compounds, including pesticides and pharmaceuticals, inhibit AChE (Holmstedt 1959). Metabolites of organophosphates (OPs), such as chlorpyrifos-oxon and diazinon-oxon, can bind irreversibly to AChE, thereby forming a stable adduct and preventing the hydrolysis of ACh, which results in clinical symptoms such as muscle weakness, paralysis, coma, or even death (Richardson et al. 2019). Other classes of pesticides, like carbamates (e.g. pirimicarb and carbaryl) can bind reversibly to AChE and can cause the same types of (clinical) symptoms as OP-poisoning, but will not result in chronic toxicity as the bonds will hydrolyse spontaneously within a few hours, resulting in fully functioning AChE again (Fukuto 1990). AChE is also inhibited by pharmaceutical carbamates like rivastigmine, which is used in the treatment of Alzheimer’s disease (Pinho et al. 2013). Besides its importance for the nervous system, AChE is also present in the blood, where it is involved in the nitric oxide signal pathway (Saldanha 2017). As measuring human AChE activity in the nervous system is difficult, red-blood cell AChE activity is often taken as a surrogate biomarker for AChE activity in the nervous system. Multiple studies confirm that there is a high functional similarity between the two and that measuring blood AChE activity is a fast and easy way to assess AChE activity in the nervous system (Duncan and Griffith 1992; Worek et al. 2012).

**Fig. 1:**
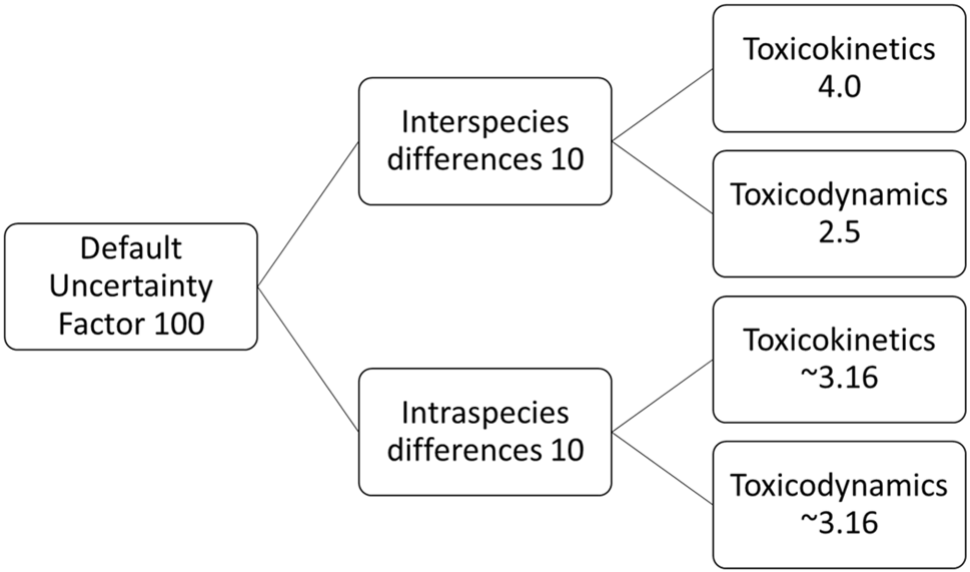
Schematic overview of the default uncertainty factors used to allow for interspecies and intraspecies differences in toxicokinetics and toxicodynamics

In chemical risk assessment, a 100-fold uncertainty factor (UF) is applied to extrapolate from a safe dose in animals, such as the no observed adverse effect level (NOAEL), to a safe level of exposure in humans (Dorne and Renwick 2005). This factor is divided into two factors of 10, one that accounts for interspecies differences and one that accounts for human variability (intraspecies differences). These factors of 10 are again divided into two factors, to account for variability in toxicokinetics (TK) and for variability in toxicodynamics (TD) (Fig. 1; Renwick and Lazarus 1998). Although this default UF of 100, also referred to as the 100-fold margin of safety, was introduced over 60 years ago (Lehman and Fitzhugh 1954) and is still used today, it is very unlikely that this default UF is appropriate for all chemicals. The UFs should allow for differences in responses between all laboratory animal species and humans and cover the entire range of human variability, regardless of the kinetic and dynamic properties of a chemical (Walton et al. 2001). Therefore, efforts are made to refine the default UFs and work towards pathway-specific or chemical-specific adjustment factors (CSAFs) (Bhat et al. 2017; IPCS 2005; Renwick et al. 2001).

In vitro methods can contribute to the refinement of the default UFs, while complying with the 3Rs principle of the reduction, refinement and replacement of animal testing (Russell and Burch 1959). Although the use of an interspecies differences UF may be eliminated when human cells and/or tissue are used in the transition towards an alternative risk assessment without animal experiments, it can still be useful to investigate these interspecies differences to gain confidence in the alternative method used and compare with an animal in vivo data (Punt et al. 2020). To refine the UF for human variability, i.e. intraspecies differences, cells or tissues from different human donors can be used. Furthermore, the use of in vitro assays can be useful in deriving pathway-related or chemical-specific adjustment factors, particularly for assessing TD variability. A relevant example of an useful in vitro method for this purpose is the measurement of AChE inhibition. The inhibition of AChE by multiple chemicals in human donor blood can provide data on human variability measured for one endpoint with a consistent method that covers solely toxicodynamic processes. Moreover, interspecies differences can be investigated by comparing such human data with electric eel AChE, which is a convenient, well-studied source of AChE that has been often used to investigate AChE inhibition (de Jong and Wolring 1984).

In this study, we used an in vitro method to analyse inhibition of AChE as a toxicity endpoint to assess variability in TD. The aims of this study are: (1) to measure the inhibition of AChE using eight different compounds, (2) to assess interspecies differences in AChE inhibition by comparing electric eel and human AChE, (3) to assess human variability in both baseline activity and inhibition of AChE activity for future integration in physiologically based kinetic/dynamic models accounting for variability in both kinetics and dynamics. This is accomplished by assessing AChE inhibition in human donor blood and electric eel enzyme to assess the relevance of the default UF in toxicodynamics for both interspecies differences and human variability.

## Materials and methods

### AChE activity assay

#### Chemicals and solutions

Chlorpyrifos (CPF, CAS:2921-88-2), phosmet (PM, CAS:732-11-6), diazinon (DZN, CAS:333-41-5) pirimicarb (PI, CAS: 23103-98-2), rivastigmine (RI, CAS: 123441-03-2), ethopropazine (Sigma E5406), DTNB (Sigma D8130), acetylthiocholine (Sigma A5751), Triton X-100 (Sigma T8787) and *Electrophorus electricus* AChE (Sigma C2888) were all obtained from Sigma-Aldrich (Zwijndrecht, The Netherlands). Chlorpyrifos-oxon (CPO, CAS: 5598-15-2) and diazinon-oxon (DZO, CAS: 962-58-3) were obtained from Accustandard (New Haven, CT), and phosmet-oxon (PMO, CAS: 3735-33-9) was obtained from Dr. Ehrenstorfer GmbH (Augsburg, Germany).

A 50 mM sodium phosphate buffer, consisting of 6.62 g/L Na_2_HPO_4_ and 0.41 g/L NaH_2_PO_4_ in sterile water, pH adjusted to 8.0 and a 0.03% (v/v) Triton X-100 buffer in sterile water were prepared and both buffers were stored at 4 °C until use. A 1.3 mM DTNB buffer and a 6 mM acetylthiocholine buffer were prepared in the 50 mM sodium phosphate buffer, a 3 mM ethopropazine buffer was prepared in sterile water and all were stored in aliquots at − 20 °C. A 600 µM ethopropazine buffer was prepared just before use in sodium phosphate buffer. A stock solution of electric eel AChE was prepared at a concentration of 222 U/mL in 20 mM Tris–HCl buffer at pH 7.5.

Stock solutions of CP, CPO, PM, PMO, DZN, DZO (30 mM), PI (300 mM for human blood and 1350 mM for electric eel) and RI (100 mM for human blood and 1225 mM for electric eel) were all prepared in methanol and stored at − 20 °C. Stock solutions were diluted in methanol to create a dosing series and also stored at − 20 °C. These series were 100× diluted in sodium phosphate buffer to get 1% methanol solutions, resulting in a final concentration of 0.1% methanol in the wells.

#### Sample preparation and exposure

AChE from *Electrophorus electricus* (electric eel) and freshly drawn (maximum one-day-old), venous EDTA whole blood samples from healthy human donors were used. The enzyme, either from electric eel or from human blood, was exposed to sodium phosphate buffer (unexposed) or to seven different concentrations (including vehicle: 0.1% methanol) of eight different compounds (CP, CPO, PM, PMO, DZ, DZO, PI, RI) to create a full concentration–response curve. Compound, vehicle or buffer (10 µL) was added to 90 µL electric eel AChE 4.44 mU/well or to 90 µL undiluted blood in a 48-wells plate (Greiner). Following incubation for 30 min at room temperature, electric eel AChE was used directly in the enzyme activity assay (see “Enzyme activity assay”). For human blood AChE, 50 µL of the blood was added to a new 48-wells plate and 950 µL Triton buffer was added to these wells (20× dilution).

#### Enzyme activity assay

The enzyme activity assay is based on the Ellman principle, as described previously (Ellman et al. 1961; Worek et al. 1999). Briefly, 20 µL of electric eel AChE or 20 µL of Triton-diluted blood (either exposed or unexposed) and 20 µL ethopropazine were added to 460 µL of DTNB buffer in a 48-wells plate. The plate was incubated for 20 min at room temperature to allow for completion of the reaction between blood matrix thiols and DTNB, and to allow for the complete inhibition of BChE by ethopropazine. After incubation, 100 µL of acetylthiocholine was added and absorbance was read at 436 nm for 10 min using a Tecan infinite M200 spectrophotometer (Tecan Group, Ltd., Männedorf, Switzerland). Blank wells contained only DTNB buffer and unexposed enzyme/blood to correct for any background absorbance. To these wells, 100 µL sodium phosphate buffer was added instead of acetylthiocholine after incubation. The final concentration of electric eel AChE was 4 mU/well.

#### Haemoglobin determination

Unexposed, diluted blood (100 µL) was added to a 96-wells plate and 100 µL transformation agent (20 mg potassium ferricyanide, 5 mg potassium cyanide, 100 mg sodium bicarbonate and 50 µL Triton X-100 in 100 mL water) was added. This was incubated for 10 min at room temperature and the absorbance was read at 546 nm using the Tecan infinite M200 spectrophotometer. Blank wells contained transformation reagent and buffer to correct for any background absorbance.

#### Data analysis and statistics

Total haemoglobin concentration in human donor blood was calculated using the extinction coefficient, *ε*, of haemoglobin, the pathlength and the absorption value:

1 $${\text{Haemoglobin}}\, \left( {\upmu {\text{M}}} \right) = \frac{{{\text{Sample}}\, \left( {{\text{absorption}}} \right) - {\text{Blank}}\,\left( {{\text{absorption}}} \right)}}{{\varepsilon \times {\text{pathlength}}}} \times 1000 \times 1000,$$

with the pathlength equal to 0.588 cm (the height of 200 µL liquid in a 96-wells plate) and ε equal to 10.8 × 10^3^ M^−1^ cm^−1^.

The enzyme activity was calculated using the absorption value, *ε* of TNB and the pathlength:

2 $${\text{AChE activity}}\, \left( {\upmu {\text{mol}}/{\text{L}}/{\min}} \right) = \frac{{{\text{Sample}} \,\left( {{\text{absorption}}/{\min}} \right) - {\text{Blank}} \,\left( {{\text{absorption}}/{\min}} \right)}}{{\varepsilon \times {\text{pathlength}}}} \times 1000 \times 1000,$$

with the pathlength equal to 0.6 cm (the height of 600 µL liquid in a 48-wells plate) and ε equal to 10.6 × 10^3^ M^−1^ cm^−1^.

Then, enzyme activity was corrected for haemoglobin content (Worek et al. 1999):

3 $${\rm{AChE}}\;{\rm{~activity}}\,\left( {{\rm{mU}}/{\upmu \rm{mol~}}\;{\rm{Hb}}} \right) = ~\frac{{{\rm{AChE~}}\;{\rm{activity}}~\left( {\upmu{\rm{ mol}}/{\rm{L}}/{\rm{min}}} \right)}}{{{\rm{Haemoglobin~}}\left( {\upmu {\rm{M}}} \right)}} \times 15 \times 1000$$

with a factor of 15 to correct for different dilutions of blood upon determining haemoglobin concentration and AChE activity (2 times in haemoglobin determination and 30 times in AChE assay). All exposures were performed once and all measurements were performed in triplo, except for the OP parent compounds (CPF, PM, DZN), which were performed once. For electric eel AChE, Eq. (2) was used to calculate AChE activity.

All in vitro data were analysed in GraphPad Prism (version 8.4) and are expressed as mean ± standard deviation (SD). For data analysis, vehicle control (0.1% methanol) was set at 100% for both human and electric eel AChE. For human blood, the technical replicates were averaged to perform statistical tests. Unpaired *t* tests were used to test for differences between men and women and a one-way ANOVA followed by a post-hoc Tukey test was used to compare data between different age groups (group 1: ≥ 60 years; group 2: 31–60 years; group 3: ≤ 30 years). The electric eel AChE data consisted of 3 technical replicates per experiment and 3–4 independent biological replicates.

### Hierarchical Bayesian analysis

#### Literature search baseline AChE values

A horizontal literature search was performed to collect data on AChE inhibition in vitro. Data were included when AChE activity was measured in whole blood, red blood cells or plasma and when a mean and SD were reported. Most studies used the Ellman method, but other methods like the delta pH method were also included.

#### Derivation of interindividual variability in AChE baseline activity

A Bayesian hierarchical model for the meta-analysis of AChE baseline activity was implemented as described previously (Darney et al. 2019), non-informative priors were used. Uncertainty around the baseline activity was quantified using median values and 95% confidence intervals. The coefficient of variation (CV) was also estimated as follows:

4 $${\text{CV}} = \sqrt {{\exp}\left( {\ln \left( {\sqrt {{\exp}(1/\tau_{j} } } \right)} \right)^{2} - 1.}$$

Since the reported units were not standardised between the different papers, here ${\tau }_{j}$ is the inter-individual variability of the activity for a reported unit ‘*j*’.

#### Derivation of interindividual variability and uncertainty factors in AChE inhibition

The same hierarchical Bayesian model was implemented for the analysis of the in vitro data on AChE inhibition and also here non-informative priors were used. As AChE activity was tested in triplicates, donors represent the first level of the hierarchical Bayesian model and a second level for each chemical-specific AChE inhibition (‘*j*’) was used. The coefficient of variation was estimated based on Eq. (4) and uncertainty around the inhibition of activity was quantified using median values and 95% confidence intervals. UFs that cover 97.5% of the adult population were calculated as the ratio between the median of the baseline activity and the 2.5th percentile of the AChE activity after exposure.

#### Software

All the Bayesian modelling was performed in R (version 3.6) and implemented with Jags version 4.2.0 (Plummer 2003).

## Results

### Inhibition of electric eel AChE activity by eight compounds

**Fig. 2:**
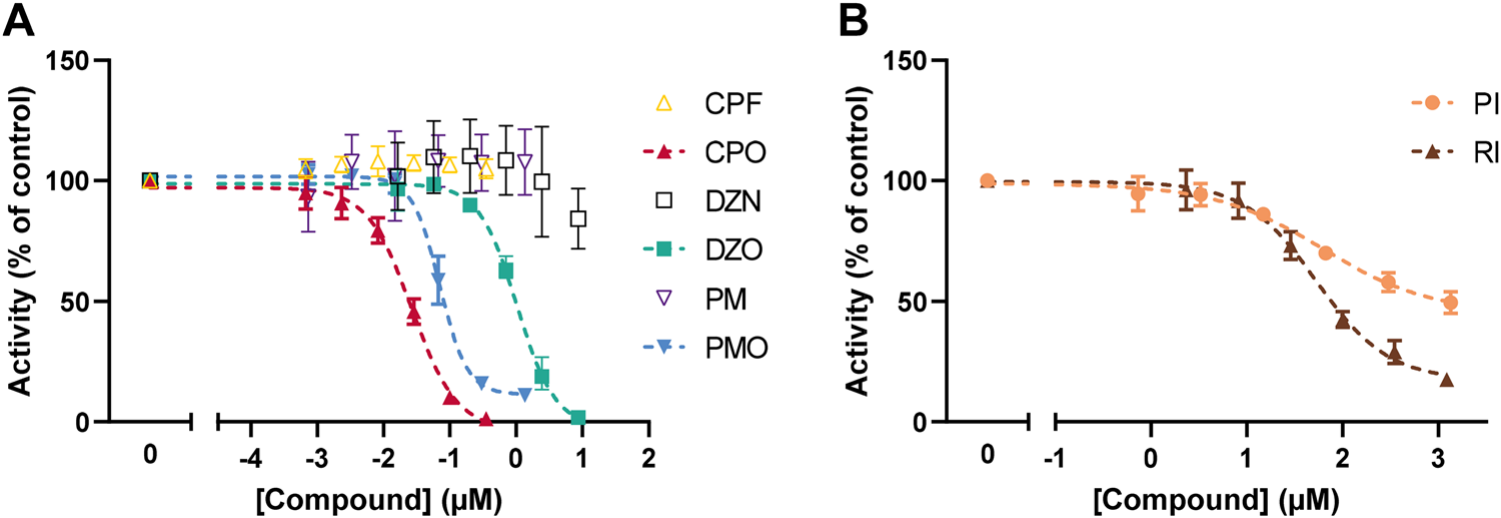
Concentration–response curves of AChE activity following exposure to eight different compounds in electric eel. **a** The OP parent compounds (CPF, DZN, PM; open symbols) do not show any AChE inhibition. The oxon metabolites of the OPs (CPO, DZO, PMO; closed symbols) show inhibition of AChE. **b** The two carbamates (PI and RI) also show inhibition of AChE. Data are presented as mean ± SD of 3–4 independent experiments

Inhibition of electric eel AChE activities measured after exposure to the eight test compounds was assessed to provide a basis to analyse interspecies differences with concentration–response curves from human donor blood. Figure 2 shows the concentration–response curves for all eight compounds. Figure 2a shows that CPF, PM and DZN are indeed far less potent than their metabolites and no inhibition of AChE is observed for these parent compounds at concentrations up to 8.4 µM. In addition, it is shown that the OP metabolites all inhibit AChE in the nM to low µM range. The IC_50_s for electric eel are 27 nM for CPO, 70 nM for PMO, and 1.03 µM for DZO (Fig. 2a). The carbamates PI and RI are less potent, with IC_50_s in the µM range: 61 and 53 µM, respectively (Fig. 2b).

## Measured haemoglobin levels and baseline AChE activity in human donor blood

Haemoglobin (Hb) levels and baseline AChE activities were determined and compared between men and women. The mean Hb level in all donors was 165 ± 16 µM and no significant differences were observed between men and women: 165 ± 20 µM vs. 164 ± 9 µM, respectively. The baseline activity of AChE for men was on average 490 ± 79 mU/µmol Hb and the average AChE activity for women was 470 ± 46 mU/µmol Hb (non-significant differences). Significant differences between age groups were not observed for either haemoglobin levels or baseline AChE activities (Supplementary Table S1).

## Inhibition of AChE activity by eight compounds in human donor blood

**Fig. 3:**
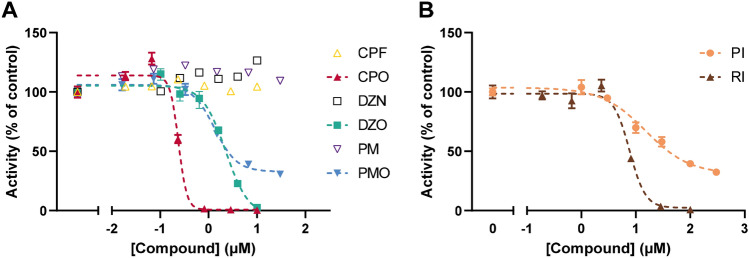
Concentration–response curves of AChE activity following exposure to eight different compounds for one human donor. **a** The OP parent compounds (CPF, DZN, PM; open symbols) do not show any AChE inhibition. The oxon metabolites of the OPs (CPO, DZO, PMO; closed symbols) show inhibition of AChE. **b** The two carbamates (PI and RI) also show inhibition of AChE. Data are presented as mean ± SD of three technical replicates of one donor for CPO, DZO, PMO, PI and RI and 1 technical replicate is depicted for CPF, DZN and PM

The blood from each donor was exposed to different concentrations of the test compounds to compare both the concentration–response curves for the different donors and for the different compounds. The results for one donor are depicted in Fig. 3. Similar to the electric eel data (Fig. 2a), CPF, PM and DZN do not inhibit AChE activity in human blood (Fig. 3a). In addition, results indicate that the OP metabolites are more potent than the carbamates. Moreover, OP metabolites are clearly more potent on electric eel AChE, whereas the carbamates (PI and RI) are more potent on human AChE. The IC_50_s for this donor can be found in Supplementary Table S1 (donor 9).

**Fig. 4:**
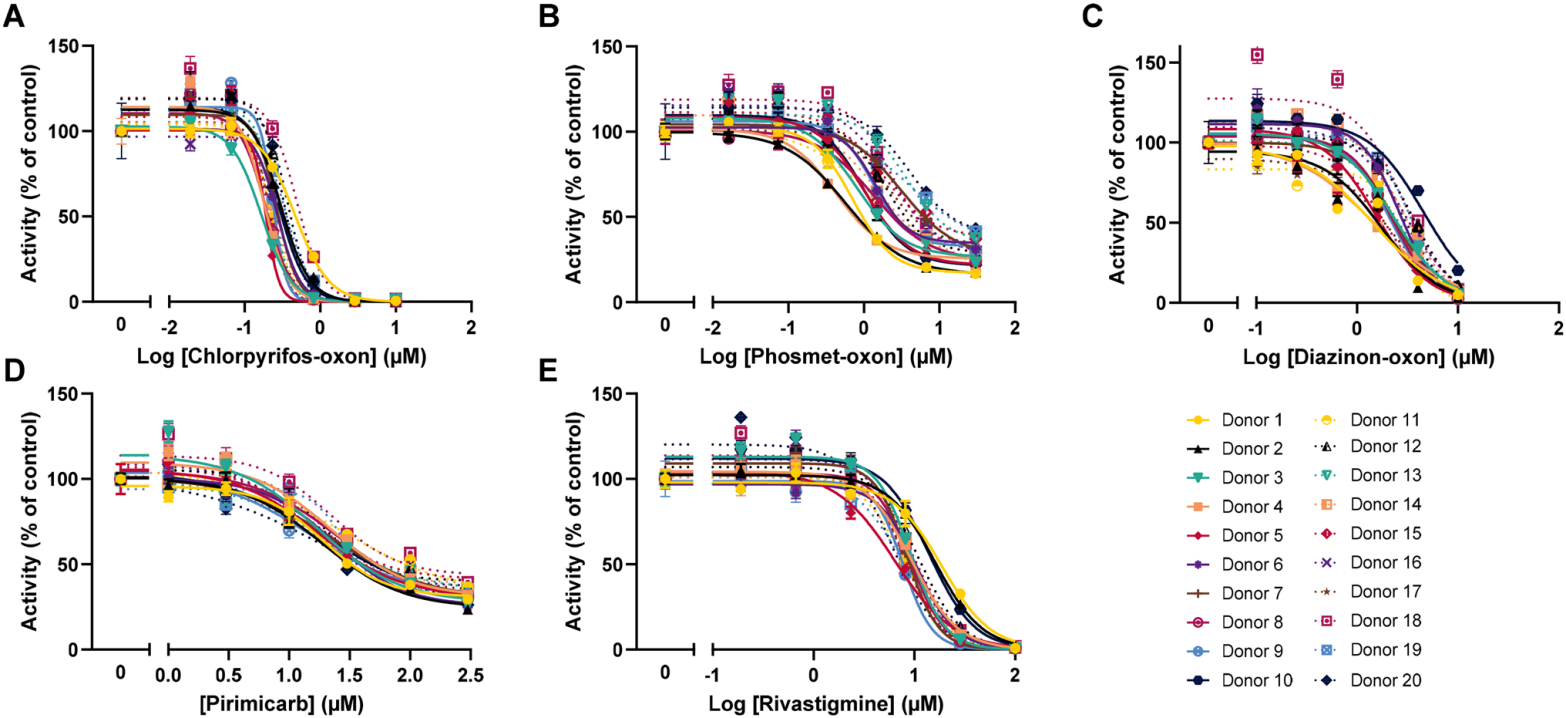
Concentration–response curves of AChE activity following exposure to the five inhibiting compounds for all (20) donors: chlorpyrifos-oxon (CPO; **a**), phosmet-oxon (PMO; **b**), diazinon-oxon (DZO; **c**), pirimicarb (PI; **d**), rivastigmine (RI; **e**). Each symbol-colour combination represents one donor. Data are presented for each donor as the percentage of vehicle control AChE activity for that donor (mean ± SD of three technical replicates)

The concentration–response curves for all donors after exposure to CPF, PM and DZN are depicted in Supplementary Figure S1. Figure 4 depicts the concentration–response curves for all donors and the five compounds that inhibit AChE. The average IC_50_s for all donors is 0.3 ± 0.1 µM for CPO, 1.9 ± 1.1 µM for PMO, 2.5 ± 0.8 µM for DZO, 20 ± 4.3 µM for PI and 9.9 ± 3.1 µM for RI. The curves in Fig. 4 demonstrate that interindividual variability is in general low, although there are some differences between compounds. It can also be derived that in general for each donor, the ranking according to potency (CPO > PMO > DZO > RI > PI) is comparable. However, for some donors, the ranking is different and DZO is more potent than PMO when considering IC_50_s (Supplementary Table S1). Also ranking according to IC_20_s resulted in a reversed order of potency for PI and RI for some donors (donor 2, 9, 10 13 and 17).

## Comparison between human donors and interindividual variability

To estimate variability in baseline AChE activity in the human population, a meta-analysis was performed using data from the  literature. Overall, variability in baseline AChE activity was 16% (CV), calculated from 19 studies and 4950 individuals. In the current in vitro study, variability in baseline AChE activity was 4.1–6.5% (Table 1, C0).

**Table 1:** Summary of interspecies and interindividual differences in AChE inhibition

Chemical	Inhibitory constants				Coefficient of variation (CV)							Human uncertainty factor (UF)
	IC_50_	IC_20_	IC_50_	IC_20_	C0	C1	C2	C3	C4	C5	C6	UF_97.5_
	Electric eel		Human									
CPO	0.03	0.01	0.27	0.17	4.8	5.5	6.0	**12**	99	NA	NA	2.2 [1.9–2.7]
PMO	0.07	0.04	1.8	0.67	6.5	5.8	5.9	5.6	**7.7**	6.1	7	1.6 [1.4–1.8]
DZO	1.03	0.41	2.5	1.2	4.7	5.9	5.7	6.8	**6.1**	8.5	25	1.4 [1.2–1.6]
PI	61	9.9	20	7.1	4.1	5.8	6.0	**6.9**	5.7	5.8	6.6	1.8 [1.5–2.1]
RI	53	16	9.9	5.1	6.0	5.5	6.0	5.8	**8.6**	25	300	2.0 [1.7–2.4]

Inhibition constants derived from the concentration–response curves for electric eel and the average of 20 human donors are depicted in µM (IC_50_ and IC_20_ values) for the five compounds that showed inhibition of AChE: chlorpyrifos-oxon (CPO), phosmet-oxon (PMO), diazinon-oxon (DZO), pirimicarb (PI) and rivastigmine (RI). Moreover, coefficients of variation (CV, in %) for each concentration are depicted to indicate human variability in AChE inhibition. The associated uncertainty factor (UF_97.5_) for interindividual differences with the 95% confidence interval (95% CI) for the concentration closest to the IC_20_ (in bold) is illustrated. NA: not estimated; C0-6: concentration level from 0 (C0) to the highest concentration (C6), the corresponding concentrations for each chemical are depicted in Supplementary Table S2

To characterise inter-individual variability in AChE inhibition from the current in vitro study, the IC_50_s and IC_20_s for all donors were assessed and compared, and differences between sexes, age groups and compounds were examined (Supplementary Table S1). IC_20_s are reported here in addition to IC_50_s as inhibition of AChE ≥ 20% is already considered adverse (Carlock et al. 1999; EFSA 2019). No sex differences across the different compounds were observed for the inhibition of AChE activity. Moreover, for all compounds, no significant differences were observed between the three different age groups for both IC_50_s and IC_20_s.

Measured IC_50_s and IC_20_s are reported in Table 1 for both electric eel and human AChE. Interindividual variability across humans was estimated for each chemical and tested concentration. For CPO, the most potent AChE inhibitor, variability could not be estimated for the two highest concentrations. CPO inhibits AChE activity completely at concentrations ≥ 2.9 µM and interindividual variability cannot be estimated when measured activities are 0. Overall, interindividual variability ranged between 5–8% and reached 25% with the highest DZO concentration and up to 300% with the highest RI concentration. This is due to some activity measurements being equal to 0 at the highest concentration of RI (100 µM; range: 0–13 mU/µmol haemoglobin). The UF_97.5_ regarding interindividual variability was calculated as the ratio between the median of the control AChE activity and the 2.5th percentile of inhibition for the concentration closest to the IC_20_ (Supplementary Table S2). The UF is highest for CPO (2.2) and lowest for DZO (1.4) and all UFs (including 95% confidence interval) are below the default UF of ~ 3.16.

## Discussion

This manuscript provides an assessment of interspecies and interindividual differences in AChE inhibition resulting from exposure to eight compounds, including seven pesticides and a pharmaceutical. Three compounds did not inhibit AChE (CPF, PM, DZN) and the other five compounds inhibited AChE, with the OP-oxons clearly being more potent than carbamates. Although this is the first study that assessed inhibition of AChE by several compounds using blood from multiple human donors, the derived IC_50_s are generally close to previously reported IC_50_s using other sources of (human) AChE. Similarly, derived electric eel IC_50_ values are close to those reported previously (Table 2). No in vitro studies on AChE could be identified for PMO and PI (EFSA 2005, 2011), indicating that this is the first study addressing in vitro AChE inhibiting potency for these compounds in humans. In addition, the concentrations used in this study for all compounds were relevant to the human in vivo situation (Table 2).

**Table 2:** Comparison of inhibition constants (IC_50_s) from this study and IC_50_s reported in literature

	Electric eel		Human				
Chemical	IC_50_ Value	References	Source	IC_50_ Value	Reference	In vivo concentration	Reference
CPO	*0.03*	*This study*	*Blood*	*0.27*	*This study*	≤ 0.06	Eyer et al. (2009)
	0.03	Čolović et al. (2011)	Recombinant enzyme	0.35	Sipes et al. (2013)	≤ 0.02	Heilmair et al. (2008)
	0.01	Jett et al. (1999)	Recombinant enzyme	0.014	Li et al. (2019)		
	0.01	Meijer et al. (2014)					
PMO	*0.07*	*This study*	*Blood*	*1.8*	*This study*		
DZO	*1.03*	*This study*	*Blood*	*2.5*	*This study*		
	0.05	Čolović et al. (2011)	Recombinant enzyme	1.3	Li et al. (2019)		
PI	*61*	*This study*	*Blood*	*20*	*This study*	≤ 300	Hoffmann et al. (2008)
RI	*53*	*This study*	*Blood*	*9.9*	*This study*	≤ 0.34	Lefèvre et al. (2016)
	11	Uysal et al. (2018)	Neocortex	9.1	(Jackisch et al. 2009)	~ 0.1	Hossain et al. (2002)
	11	Tehrani et al. (2019)					
	56	(Kratky et al. 2018)					
	501	(Imramovsky et al. 2012)					
CPF	*> 0.35*	*This study*	*Blood*	*> 10*	*This study*	≤ 10	Buratti et al. (2003)
	4.5	Čolović et al. (2011)	Blood	0.12	Das et al. (2006)	≤ 5	Huen et al. (2012)
	0.03	(Assis et al. 2012)	Red blood cells	200	(Ajilore et al. 2018)	≤ 5	Eyer et al. (2009)
PM	*> 1.4*	*This study*	*Blood*	*> 30*	*This study*	≤ 50	Santori et al. (2020)
			Recombinant enzyme	Inactive	(Li et al. 2019)		
DZN	*> 8.5*	*This study*	*Blood*	*> 10*	*This study*	≤ 10	Buratti et al. (2003)
	> 200	Čolović et al. (2011)	Recombinant enzyme	38	Li et al. (2019)		
	0.3	Assis et al. (2012)					

IC_50_s for both electric eel and human (average of all donors) are depicted for all compounds tested in this study: chlorpyrifos-oxon (CPO), phosmet-oxon (PMO), diazinon-oxon (DZO), pirimicarb (PI), rivastigmine (RI), chlorpyrifos (CPF), phosmet (PM), diazinon (DZN). For human values, the source of the enzyme is depicted. The values derived in the current study are indicated in italic and all concentrations are in µM. In vivo concentrations depict concentrations as measured or estimated in plasma following human exposure

Although AChE enzymes are well conserved in evolution (Wiesner et al. 2007), interspecies differences in activity and inhibition do exist. However, such differences are compound-specific, which is clear from the IC_50_s and IC_20_s reported here for electric eel and human AChE (Table 1). Electric eel was more sensitive compared to human AChE for CPO, PMO and DZO, whereas the carbamates were less potent in electric eel AChE. Such species differences between birds, fishes and mammals, including rats, have also been demonstrated in earlier reports for brain AChE inhibition, particularly for oxon compounds and carbamates (Andersen et al. 1977; Chattopadhyay et al. 1986; Johnson and Wallace 1987; Murphy et al. 1968; Qadri et al. 1994). It has been hypothesised that differences in affinity and the rate of phosphorylation of AChE or differences in the steric arrangement in the active site can contribute to species differences, resulting in different IC_50_s and species sensitivity distributions dependent on the compound (Andersen et al. 1977; Wang and Murphy 1982). Consequently, a mechanism- or toxicodynamic-specific adjustment factor is unsuitable for assessing interspecies differences related to AChE inhibition. Instead, chemical-specific adjustment factors should be considered, given the chemical dependency of the adjustment factor. Although electric eel AChE can provide a quick and easy screening tool for hazard identification, it is important to note that for some of the tested compounds the default UF for interspecies differences in toxicodynamics (2.5) is insufficient for AChE inhibition. Moreover, since rat data is most often used in risk assessments for these chemicals and interspecies differences are large, the use of electric eel as a useful in vitro method for risk assessments is questionable and rat or human data may be preferred.

The modified Ellman method is a sensitive method to determine AChE activity (Ellman et al. 1961; Worek et al. 1999). Notably, it is important to use fresh blood for performing these experiments with this method. Although several studies demonstrated no decline in AChE activity up to 2 months after freezing (Naik et al. 2013; Worek et al. 1999), freezing the blood at − 20 °C resulted in unreliable measurements with much more technical variability than experiments performed with fresh (maximum one-day-old, stored at 4 °C) blood (data not shown). Another important aspect of using this method, is to distinguish AChE from BChE in the blood, as BChE can also bind to the test compounds, thereby limiting AChE inhibition. This is accomplished here by adding the specific BChE inhibitor ethopropazine to the blood (Naik et al. 2013).

The meta-analysis showed an interindividual variability in AChE baseline activity of 16%, which is higher than the variability reported here for the vehicle controls (4.1–6.5%, Table 1). This is most likely due to the fact that the meta-analysis included data reported in different peer-reviewed papers which use different methods, resulting in interlaboratory and interstudy variability in contrast to the consistent method applied here. Baseline values of AChE activity measured in the current study (average 482 mU/µmol Hb) were close to activities reported in Worek et al. (1999) (586–651 mU/µmol Hb) and Karasova et al. (2017) (351 ± 67 for men and 377 ± 65 for women). A significant difference in baseline activity between men and women has been previously reported, with women having slightly higher activity values than men (Karasova et al. 2017). In the current study, no significant sex differences were observed, which may be due to the smaller sample size. Furthermore, Karasova et al. (2017) reported no significant effect of age on baseline AChE levels, which was confirmed in the current study.

We evaluated human variability in AChE inhibition to assess human variability in TD. This variability represents solely variability in TD, as ADME processes are not involved when using human donor blood. Although paraoxonase-1 (PON1) is present in blood and involved in the metabolism of some of the compounds studied here, it is inactivated by EDTA (Mackness 1998). Since EDTA was added to the blood, detoxification of the OP metabolites does not occur and thus variability solely in TD was assessed. Interindividual variability in AChE inhibition was generally small. Although the UF calculated here is only 1.5–2.2 and hence considerably below default, no definite conclusions can be drawn from this yet, as the sample size is small. More research would be required to confirm this UF, but the CVs suggest limited human variability for this TD endpoint.

Consequently, the susceptibility of an individual to an OP or carbamate will mainly depend on variability in TK (Lockridge et al. 2016). We have demonstrated for the OPs that the parent compounds are far less potent in inhibiting AChE than their oxon metabolites, indicating that variability and susceptibility is at least partially dependent on the rate of formation of these metabolites. Indeed, interindividual variability in the formation of CPO after exposure to CPF is extensive (Eyer et al. 2009) and is ethnicity-dependent (Zhao et al. 2019). Also for PON1-mediated detoxification, pathway-specific UFs and the corresponding interindividual and interphenotypic variability have been assessed previously and have proven to be higher than the default UF of ~ 3.16 for some genotypes (Darney et al. 2020). Notably, to fully acknowledge interindividual variability for chemicals, information on both kinetics and dynamics should be considered. Therefore, incorporation of interindividual variability in toxicodynamic endpoints such as AChE inhibition into physiologically-based models alongside kinetic variability (like for PON1) can help refine risk assessment of chemicals using benchmark dose approaches to derive health-based guidance values within a 3R framework (EFSA Scientific Committee et al. 2017).

In in vivo human situations, toxicokinetics are always involved and are often a major contributor to human variability. It is therefore hard to distinguish the individual contribution of kinetic and dynamic processes to the total variability in vivo. Our in vitro study shows a fast and easy way to assess both interspecies differences and human variability in toxicodynamics using inhibition of AChE from electric eel and in human donor blood. The interspecies differences for OP metabolites are large (up to 20 times). However, electric eel AChE is for all OP metabolites more sensitive compared to human AChE, suggesting that no UF is necessary to correct for interspecies differences to ensure human safety for these compounds when electric eel AChE is used as a point of departure for risk assessment. On the other hand, humans are more sensitive to the carbamates and the default UF of 2.5 seems to be insufficient to cover the interspecies differences between electric eel and humans. In contrast, human variability was small both for both OP metabolites and carbamates and the default toxicodynamic UF of ~ 3.16 for appears to be sufficient to cover such human variability for concentrations of OP metabolites and carbamates that inhibit ~ 20% of AChE activity. Combined, our in vitro data can support the improvement of risk assessment and derivation of toxicodynamic-related and chemical-specific uncertainty factors. Furthermore, the observed interspecies differences highlight the importance of the transition towards the use of human in vitro models as alternatives to animal models for hazard identification and characterisation.

## Electronic supplementary material

Below is the link to the electronic supplementary material.

Supplementary file1 (DOCX 515 kb)

## Data Availability

The datasets generated during and/or analysed during the current study are available from the corresponding author on reasonable request.

## Code availability

The codes used during the current study are available from the corresponding author on reasonable request.
