# Supplementary material for: Acetylcholinesterase inhibition in electric eel and human donor blood: an in vitro approach to investigate interspecies differences and human variability in toxicodynamics
Source: Arch Toxicol. 2020 Oct 10;94(12):4055–65. doi: 10.1007/s00204-020-02927-8 (PMC7655571; doi:10.1007/s00204-020-02927-8)
Supplement: Supplementary file 1 — Supplementary file1 (DOCX 515 kb) [file 204_2020_2927_MOESM1_ESM.docx]

**Supplementary Information:**

**Acetylcholinesterase inhibition in electric eel and human donor blood: an *in vitro* approach to investigate interspecies differences and human variability in toxicodynamics**

Emma E.J. Kasteel^1^, Sandra M. Nijmeijer^1^, Keyvin Darney^2^, Leonie S. Lautz^2^, Jean Lou C.M. Dorne^3^, Nynke I. Kramer^1^, Remco H.S. Westerink^1,#^

^1^Institute for Risk Assessment Sciences (IRAS), Toxicology Division, Faculty of Veterinary Medicine, Utrecht University, P.O. Box 80.177, 3508TD, Utrecht, The Netherlands

^2^Risk Assessment Department, French Agency for Food, Environmental and Occupational Health & Safety (ANSES), 14 rue Pierre et Marie Curie, Maisons-Alfort, F-94701, France

^3^European Food Safety Authority, Scientific Committee and Emerging Risks Unit, Via Carlo Magno 1A, 43126 Parma, Italy

^#^Corresponding author: [r.westerink@uu.nl](mailto:r.westerink@uu.nl)

Journal name: Archives of Toxicology

**Figure S1.** Concentration-response curves of AChE activity following exposure to the three negative control compounds for all human blood donors: chlorpyrifos (CPF; A), phosmet (PM; B) and diazinon (DZN; C). Each symbol-colour combination represents one donor. Data are for each donor presented as the percentage of vehicle control AChE activity for that donor.

**Table S1.** Inhibition constants derived from the concentration-response curves for all human blood donors. Characteristics of the donor (birth year, sex, haemoglobin level) and inhibition constants (IC_50_ and IC_20_ values, in µM) including 95% Confidence Intervals (CI) are listed for the five compounds that showed a potent inhibition of AChE: chlorpyrifos-oxon (CPO), phosmet-oxon (PMO), diazinon-oxon (DZO), pirimicarb (PI) and rivastigmine (RI). M: male, F: female. ?: Confidence intervals could not be calculated.

| Donor | Birth year | Sex | Haemoglobin (µM) | µM | CPO | | PMO | | DZO | | PI | | RI | |
| --- | --- | --- | --- | --- | --- | --- | --- | --- | --- | --- | --- | --- | --- | --- |
|  |  |  |  |  | Value | 95% CI | Value | 95% CI | Value | 95%CI | Value | 95%CI | Value | 95%CI |
| 1 | 1952 | M | 153 | *IC_50_* | 0.5 | [0.4-0.5] | 0.7 | [0.6-0.8] | 1.5 | [1.0-2.1] | 19 | [15-26] | 19 | [16-22] |
|  |  |  |  | *IC_20_* | 0.2 | [0.2-0.3] | 0.3 | [0.3-0.4] | 0.5 | [0.2-0.9] | 8.6 | [5.5-12] | 8.6 | [6.5-11] |
| 2 | 1972 | F | 155 | *IC_50_* | 0.3 | [0.3-0.4] | 0.6 | [0.5-0.6] | 1.7 | [1.1-2.6] | 19 | [15-25] | 16 | [15-17] |
|  |  |  |  | *IC_20_* | 0.2 | [0.1-0.2] | 0.2 | [0.1-0.2] | 0.7 | [0.3-1.7] | 6.4 | [4.5-8.5] | 7.6 | [6.7-8.6] |
| 3 | 1996 | M | 135 | *IC_50_* | 0.2 | [0.2-0.2] | 0.8 | [0.5-1.3] | 2.3 | [2.0-2.7] | 17 | [10-36] | 9.2 | [8.0-11] |
|  |  |  |  | *IC_20_* | 0.1 | [0.1-0.1] | 0.3 | [0.2-0.5] | 1.0 | [0.8-1.3] | 5.8 | [3.2-10] | 5.3 | [4.0-6.4] |
| 4 | 1955 | F | 152 | *IC_50_* | 0.2 | [0.1-0.2] | 0.4 | [0.3-0.7] | 1.5 | [1.3-1.7] | 22 | [15-37] | 9.4 | [8.3-11] |
|  |  |  |  | *IC_20_* | 0.1 | [0.1-0.1] | 0.2 | [0.1-0.2] | 0.4 | [0.3-0.6] | 8.0 | [5.0-12] | 4.5 | [3.5-5.6] |
| 5 | 1965 | F | 157 | *IC_50_* | 0.2 | [0.2-0.2] | 1.0 | [0.7-1.4] | 1.6 | [1.3-1.8] | 17 | [12-28] | 6.4 | [5.4-7.6] |
|  |  |  |  | *IC_20_* | 0.1 | [0.1-0.1] | 0.4 | [0.2-0.6] | 0.7 | [0.5-0.8] | 5.9 | [3.7-8.9] | 2.5 | [1.9-3.3] |
| 6 | 1993 | M | 172 | *IC_50_* | 0.3 | [0.3-0.3] | 1.4 | [1.1-1.8] | 2.6 | [2.3-3.0] | 24 | [19-32] | 11 | [10-12] |
|  |  |  |  | *IC_20_* | 0.2 | [0.2-0.2] | 0.7 | [0.6-0.9] | 1.4 | [1.1-1.8] | 9.3 | [6.4-13] | 5.7 | [4.9-6.4] |
| 7 | 1958 | M | 184 | *IC_50_* | 0.3 | [0.2-0.3] | 3.1 | [2.1-5.4] | 2.4 | [2.3-2.6] | 23 | [17-37] | 8.5 | [7.7-9.4] |
|  |  |  |  | *IC_20_* | 0.2 | [0.1-?] | 1.0 | [0.6-1.5] | 1.2 | [1.0-1.4] | 6.4 | [4.3-9.1] | 5.1 | [4.2-5.8] |
| 8 | 1995 | M | 158 | *IC_50_* | 0.2 | [0.2-0.2] | 1.3 | [1.0-2.0] | 2.2 | [2.1-2.4] | 26 | [19-38] | 9.2 | [8.3-10] |
|  |  |  |  | *IC_20_* | 0.1 | [0.1-0.2] | 0.4 | [0.3-0.6] | 1.2 | [1.0-1.3] | 8.1 | [5.6-11] | 5.3 | [4.3-6.2] |
| 9 | 1971 | M | 182 | *IC_50_* | 0.2 | [0.2-0.3] | 1.3 | [1.1-1.7] | 2.1 | [1.8-2.4] | 15 | [10-24] | 7.8 | [6.9-8.5] |
|  |  |  |  | *IC_20_* | 0.2 | [0.1-0.2] | 0.6 | [0.4-0.8] | 1.1 | [0.8-1.3] | 4.2 | [2.7-6.3] | 4.8 | [4.1-5.3] |
| 10 | 1960 | M | 128 | *IC_50_* | 0.3 | [0.2-0.3] | 1.0 | [0.8-1.3] | 4.5 | [3.6-5.7] | 20 | [13-34] | 14 | [12-17] |
|  |  |  |  | *IC_20_* | 0.2 | [0.1-0.2] | 0.4 | [0.3-0.6] | 1.9 | [1.2-2.8] | 5.3 | [3.2-8.2] | 6.8 | [5.2-8.8] |
| 11 | 1955 | F | 157 | *IC_50_* | 0.2 | [0.2-0.2] | 1.5 | [1.0-2.5] | 2.9 | [2.1-?] | 29 | [16-93] | 8.0 | [6.8-9.3] |
|  |  |  |  | *IC_20_* | 0.1 | [0.1-?] | 0.3 | [0.2-0.4] | 1.7 | [0.8-?] | 7.0 | [3.9-13] | 4.0 | [2.9-5.5] |
| 12 | 1957 | M | 176 | *IC_50_* | 0.3 | [0.3-0.4] | 1.9 | [1.4-2.8] | 3.7 | [3.2-4.2] | 18 | [14-24] | 11 | [10-12] |
|  |  |  |  | *IC_20_* | 0.2 | [0.2-0.3] | 0.8 | [0.5-1.1] | 1.9 | [1.5-2.3] | 8.8 | [5.8-13] | 5.6 | [4.6-6.6] |
| Donor | Birth year | Sex | Haemoglobin (µM) | µM | CPO | | PMO | | DZO | | PI | | RI | |
|  |  |  |  |  | Value | 95% CI | Value | 95% CI | Value | 95%CI | Value | 95%CI | Value | 95%CI |
| 13 | 1995 | M | 186 | *IC_50_* | 0.2 | [0.2-0.3] | 3.7 | [2.3-6.6] | 2.5 | [2.3-2.8] | 19 | [13-35] | 10 | [8.9-10] |
|  |  |  |  | *IC_20_* | 0.2 | [0.1-0.2] | 1.5 | [0.9-2.6] | 1.1 | [1.0-1.3] | 4.7 | [2.9-7.1] | 5.4 | [4.7-6.1] |
| 14 | 1957 | F | 174 | *IC_50_* | 0.2 | [0.2-0.3] | 1.4 | [0.9-2.6] | 2.8 | [2.4-3.4] | 15 | [9.5-25] | 10 | [9.1-12] |
|  |  |  |  | *IC_20_* | 0.2 | [0.1-0.2] | 0.4 | [0.2-0.7] | 1.3 | [1.0-1.7] | 6.1 | [3.7-9.4] | 5.8 | [4.7-6.9] |
| 15 | 1959 | M | 158 | *IC_50_* | 0.2 | [0.2-0.2] | 1.8 | [1.1-3.6] | 1.9 | [1.8-2.1] | 16 | [11-28] | 8.4 | [7.7-9.1] |
|  |  |  |  | *IC_20_* | 0.1 | [0.1-0.2] | 0.6 | [0.4-1.0] | 0.9 | [0.8-1.0] | 6.0 | [3.6-9.3] | 4.7 | [3.9-5.6] |
| 16 | 1970 | M | 192 | *IC_50_* | 0.2 | [0.2-0.2] | 2.3 | [1.4-4.3] | 3.0 | [2.3-3.8] | 14 | [9.7-24] | 9.3 | [7.8-11] |
|  |  |  |  | *IC_20_* | 0.2 | [0.1-0.2] | 0.8 | [0.5-1.4] | 1.3 | [0.8-2.1] | 5.9 | [3.6-8.9] | 4.9 | [3.5-6.4] |
| 17 | 1992 | M | 162 | *IC_50_* | 0.2 | [0.2-0.2] | 2.1 | [1.5-3.1] | 2.2 | [1.7-2.7] | 16 | [12-21] | 8.4 | [7.7-9.1] |
|  |  |  |  | *IC_20_* | 0.1 | [0.1-?] | 0.9 | [0.6-1.2] | 0.9 | [0.5-1.3] | 4.8 | [3.5-6.4] | 4.8 | [4.0-5.6] |
| 18 | 1976 | F | 174 | *IC_50_* | 0.5 | [0.4-0.6] | 2.3 | [1.5-4.3] | 2.9 | [2.0-4.2] | 22 | [13-55] | 9.4 | [7.7-11] |
|  |  |  |  | *IC_20_* | 0.3 | [0.2-0.4] | 1.0 | [0.6-1.7] | 1.4 | [0.8-2.3] | 9.2 | [4.8-17] | 4.6 | [3.2-6.1] |
| 19 | 1966 | F | 165 | *IC_50_* | 0.3 | [0.3-0.4] | 4.2 | [2.6-12] | 3.4 | [3.1-3.8] | 28 | [23-37] | 7.6 | [6.2-9.1] |
|  |  |  |  | *IC_20_* | 0.2 | [0.2-0.2] | 1.1 | [0.7-1.7] | 1.9 | [1.6-2.2] | 18 | [11-?] | 3.3 | [2.3-4.6] |
| 20 | 1963 | F | 173 | *IC_50_* | 0.4 | [?-0.5] | 4.0 | [2.1-16] | 2.7 | [2.3-3.2] | 19 | [10-82] | 6.1 | [4.7-7.8] |
|  |  |  |  | *IC_20_* | 0.2 | [0.2-0.3] | 1.5 | [0.7-3.0] | 1.3 | [1.0-1.8] | 3.4 | [1.7-6.4] | 3.0 | [1.9-4.7] |

**Table S2.** Concentrations used for determining AChE inhibition *in vitro* using human donor blood for chlorpyrifos-oxon (CPO), phosmet-oxon (PMO), diazinon-oxon (DZO), pirimicarb (PI) and rivastigmine (RI). In black are the concentrations closest to the calculated IC_20_s, which were used to calculate the uncertainty factors.

| Concentration | CPO | PMO | DZO | PI | RI |
| --- | --- | --- | --- | --- | --- |
| C0 | 0 | 0 | 0 | 0 | 0 |
| C1 | 0.019 | 0.016 | 0.10 | 1 | 0.19 |
| C2 | 0.067 | 0.073 | 0.26 | 3 | 0.67 |
| C3 | 0.23 | 0.33 | 0.64 | 10 | 2.33 |
| C4 | 0.82 | 1.48 | 1.6 | 30 | 8.16 |
| C5 | 2.86 | 6.67 | 4.0 | 100 | 28 |
| C6 | 10 | 30 | 10 | 300 | 100 |
